# Differences in Physical Characteristics of the Lower Extremity and Running Biomechanics Between Different Age Groups

**DOI:** 10.3390/ijerph19074320

**Published:** 2022-04-04

**Authors:** Jongbin Kim, Sang-Kyoon Park

**Affiliations:** 1 Division of Kinesiology, Silla University, Busan 46958, Korea; kjb36@silla.ac.kr; 2 Motion Innovation Center, Korea National Sport University, Seoul 05541, Korea

**Keywords:** bone mineral density, maximal strength of lower joint, aging, running, kinematics

## Abstract

(1) Background: The objective of this study was to determine physical and biomechanical changes in age groups upon running. (2) Method: 75 male adults (20–80s) participated in the study. Bone mineral density and lower extremity joint strength were measured according to age-increase targeting. Based on age, correlations among running characteristics, impulse, impact force, maximum vertical ground reaction force, loading rate, lower extremity joint 3D range of motion, joint moment, and power upon running motion were calculated. (3) Result: Older runners tended to show lower bone mineral density, extremity maximum strength, stride time, and stride distance, with smaller RoM and joint power of ankle and knee joints in the sagittal plane, compared with younger subjects. However, there were no significant correlations between age and impact variables (i.e., impulse, impact force, peak GRF, and loading rate) during running. (4) Conclusion: Older runners tend to show weaker physical strength characteristics, such as bone mineral density and muscle strength and lower joint functionality of ankle and knee joints during running, compared with younger runners. Therefore, strengthening the lower extremity muscle and improving dynamic joint function, especially for ankle joints, can be helpful for injury prevention during running.

## 1. Introduction

Physical aging causes high blood pressure, diabetes, obesity, and bone mineral density (BMD) decrease; thus, physical changes such as aerobic capacity decline, coordination decrease, muscle function weakening, decreased gait ability, and risk of chronic disease are induced [1,2,3]. Among the changes in physical characteristics due to aging, BMD gradually decreases from 35 years of age, and osteoporosis is caused from 50 years of age [4]. Muscle volume starts to decrease by 10% compared with people in their 20s and this decrease is accelerated from 65 years of age. At 70 years of age, the mean strength is 60% of that of people in their 20s [5]. Consequently, independent life becomes impossible due to fracture damage and mental health, and all this may work as a potential factor for life quality decline [6,7,8]. To delay physical aging, regular exercise is essential. Regular exercise furthers functional physical health, including physical strength retention and enhancement, cardiovascular function improvement, muscle strength increase, flexibility increase, and mental health. Likewise, regular runs are effective for health enhancement and aging prevention [9,10,11,12,13].

As interest in running has recently increased for health enhancement, participants in running races of various distances and courses have also steadily increased. Because running’s temporal, spatial, and cost limitations are slight, its universality is proven as a health enhancement exercise, compared to other physical activities [14]. Regular physical exercise and compound exercise are suitable methods to maintain BMD through proper physical activities in everyday life, and they are frequently used for BMD improvement and osteoporosis treatment [15]; however, bone health may be negatively affected [16]. BMD increase between the lumbar and the thigh showed no significant difference in endurance running but exhibited a remarkable variance in weight in a study targeting long-distance runners aged 18 to 44 [17]. Various types of studies are required to analyze the effects of exercises on BMD. According to physical aging, the decreased rate of lower extremity strength is higher than the upper extremity strength. The muscle thickness of the gastrocnemius and thigh skeletal muscles decreases as one gets older, and consequently, joint power gradually reduces [18,19,20]. The lower extremity strength is maintained and increases with resistance exercise and weight-bearing (load) exercise [21,22]. Previous studies reported an improvement of 27% total muscle strength, 27% knee joint flexion, and 17% extension strength among elderly people who performed lower extremity resistance exercises for 14 weeks [23]. The elderly’s ankle and knee joints’ flexion and extension force was 70–80% of that of male adults in general. Regular physical activities to reduce risk factors, revealed due to physiological aging, were found adequate for the retention of and delayed decrease in bone mineral and strength [24].

A study on running motions reported that sagittal plane movements impact absorption, foot stability, balancing, and acceleration in the stance section when running. Additionally, most injuries, such as ankle sprain, stress fracture, backache, and muscle rupture, occurred in the stance section [25].

According to the analysis of the result, depending on age, the range of motion of the hip joint was greater, and the range of motion of the knee and ankle joints was smaller in the elderly people group than the young adult group. The impact force occurring in the initial stage stance section was larger in the young adult group compared with the elderly group [26,27,28,29,30]. In a dynamic analysis targeting adults between 18 and 60 years of age, stride decreased along with vertical ground reaction force, ankle moment, and power [31]. If the impact was not absorbed by adequately increasing the range of motion of the knee and ankle joints while running, it was reported that a lower extremity joint injury might occur [32]. However, very low joint stiffness caused by an increased range of motion may induce running injuries of the soft tissues and musculoskeletal system of runners’ lower extremities. Regular physical activities positively affect the lower extremity BMD and joint strength. Therefore, regular physical activities reduce musculoskeletal system injuries through a reduction in physical change due to aging [33]. However, it is insufficient to explain aging through the fragmental comparison of males in their 20s and 80s biomechanically with running motions among regular physical activities through existing previous studies. This study examined the effect of aging on physical characteristics, joint movement, and joint power, targeting males from 20 to 80 who regularly ran. The objective of this study was to determine physical and biomechanical changes in age groups upon running. It was hypothesized that older runners might compromise their lower extremities’ dynamic joint function during running due to declined physical characteristics compared with younger runners.

## 2. Materials and Methods

### 2.1. Participants

The participants in this study were 75 healthy males who did not receive treatment and had surgery history due to lower extremity musculoskeletal system problems within the past six months, who ran 10 km or more three times a week, who had participated at least once in a marathon race, and whose right leg was their dominant leg (Table 1). All the participants voluntarily participated in this study. The experiment was carried out after gaining approval from the IRB (20180611-046).

### 2.2. Procedure

**Figure 1:**
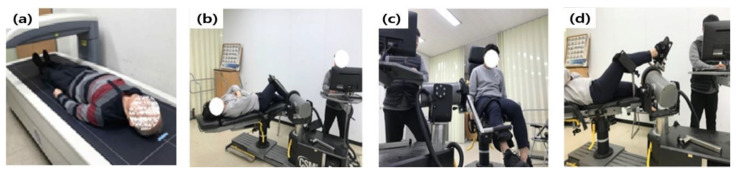
Measurements of BMD and maximum joint strength: (**a**) BMD, (**b**) hip, (**c**) knee, and (**d**) ankle joint.

After explaining the procedure and purpose of this study to the participants, they consented to the test. To collect body composition information, their height and weight were measured. The participants’ thighs and ankles were secured with a fixing band in a state where they did not move once lying down on the examination table and looking at the ceiling, with their lower extremity joint BMD being measured using the Dual Energy X-ray Absorptiometry (DEXA, QDR-1000; Hologic, Waltham, MA, USA) equipment. Their chest, abdomen, and thigh were held with a fixing band to measure their maximum lower extremity joint strength. There was no movement in the other joints except in the observed joints upon matching the dynamometer axis and joint using isokinetic exercise (Humac Norm, Stoughton, MA, USA), as shown in Figure 1 [34]. The hip joint extension (gluteus maximus) and flexion (iliopsoas), knee joint extension (quadriceps) and flexion (hamstrings), and ankle joint dorsiflexion (tibialis anterior) and plantar flexion (gastrocnemius) were measured once, setting the angle and speed at 60°/s each time. In doing so, practices were performed three times, and peak torque was measured five times. To prevent fatigue, break time was given between measurements, while a loud voice offered motivation to exert maximum strength upon measurement.

**Figure 2:**
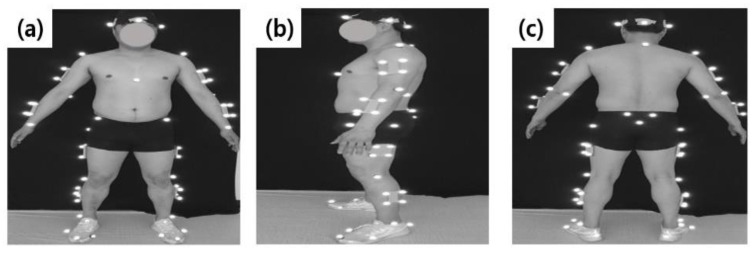
Reflective markers on the body: (**a**) frontal view, (**b**) lateral view, (**c**) backward view.

**Figure 3:**
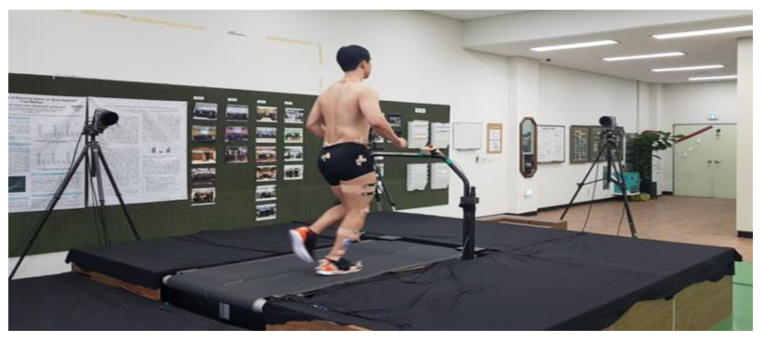
Experimental setup for running on a treadmill.

After performing a warm-up, the participants wore upper and lower tights for the motion capture test, and 64 reflective markers were attached to the body (Figure 2). They wore personal standard running shoes, and the standing calibration was carried out to ensure the participant’s body anatomical position before the running’s measurement. Eight infrared cameras (Oqus 300, Qualisys, Sweden) captured the running motions. The sampling frequency was set at 100 Hz. They ran on a treadmill embedded with two force plates (Instrumented treadmill, Bertec, corporation, Columbus, OH, USA), and the sampling rate was set at 1000 Hz. After sufficient rest to minimize muscle fatigue, measurement was carried out by gradually increasing speed for five minutes to induce natural running before the measurement. The running speed was set at 3.1 m/s [26]. During their run on the treadmill with a selected speed of five minutes, the motion was collected from 30 gait cycles of the right leg (Figure 3).

### 2.3. Data Processing

The stance phase was defined as the moment a participant’s right heel struck the toe-off on the treadmill to analyze biomechanical variables. Each joint’s position and ground reaction force data were obtained using Qualisys’s Qualisys Track Manager Program for data processing. To remove the noise of the data, Butterworth second-order low-pass filtering at 12 Hz was performed for the 3D position coordinate data [35]. The ground reaction force data were set at a power spectrum density (PSD) of 99% of the value of the cut-off frequency [36]. Using Visual 3D (C-Motion, Germantown, MD, USA) and Matlab R2014a (The Mathworks, Natick, MA, USA), the (+) joint angle from the range of motion of the ankle, knee, and hip in the sagittal plane means a flexion and dorsiflexion angle, while the (−) joint angle means an extension and plantar flexion angle. Joint moment (Nm/kg) and joint power (W/kg) (+) mean a concentric contraction, while the (–) counterpart means eccentric contraction. For the ground reaction force direction the X-axis was set as left (−) and right (+), the Y-axis was set as the front (+) and back (−), while the Z-axis was set as vertical (+).

### 2.4. Statistical Processing

For statistical processing, SPSS Ver. 25.0 software (IBM, Armonk, NY, USA) was applied. Regarding the physical characteristics, kinematics, and kinematic data obtained through the analysis program, Pearson’s product-moment correlation coefficients were calculated to find the relationship between age and physical characteristics, as well as biomechanical variables. Based on the sample size calculation, a minimum of 42 subjects was required for the expected correlation coefficient (R) of 0.35 with a power of 70% (β) [37]. A significant level of statistics was set at an alpha level of 0.05.

## 3. Results

### 3.1. Correlations between Age and Physical Characteristics (BMD) and Peak Torque

According to the correlation analysis between age and BMD, statistical significance was displayed among the total (r = −0.380, *p* ≤ 0.001), legs BMD (r = −0.506, *p* ≤ 0.000), and T-score (r = 0.442, *p* ≤ 0.000), and it showed a negative correlation. In the lower extremity strength relationship, statistically significant negative correlations were displayed between hip joint extension (r = −0.399, *p* ≤ 0.000) and flexion (r = −0.612, *p* ≤ 0.000), knee joint extension (r = −0.535, *p* ≤ 0.000) and flexion (r = −0.525, *p* ≤ 0.000), ankle joint dorsiflexion (r = −0.407, *p* ≤ 0.000), plantar flexion (r = −0.494, *p* ≤ 0.000) (Table 2).

### 3.2. Correlations between Age and Running Characteristics and Impact

As age increased, a statistically significant negative correlation was revealed between stride time (r = −0.336, *p* ≤ 0.003) and stride distance (r = −0.536, *p* ≤ 0.000) in running characteristics (Table 2). However, there were no significant correlations between age and impact variables (i.e., impulse, impact force, GRF (ground reaction force) peak, and loading rate).

### 3.3. Correlations between Age and Joint Kinetics of the Lower Extremity Joint

According to age increase, statistical significances of correlation were found in the ranges of the ankle (r = −0.352, *p* ≤ 0.002) and knee joint motion (r = −0.361, *p* ≤ 0.000) in the sagittal plane (Table 2). In addition, ankle and knee moments (ankle dorsiflexion moment: r = 0.328, *p* ≤ 0.004, knee flexion moment: r = 0.253, *p* ≤ 0.030), as well as ankle joint power (absorption: r = −0.334, *p* ≤ 0.004, and generation: r = 0.326, *p* ≤ 0.006) in the sagittal plane, were significantly lower in older runners compared with younger runners, based on the analysis of correlations (Table 2, Appendix A; Figure A3).

## 4. Discussion

This study examined the relationship between lower extremity joint physical characteristics and running biomechanical variables and age, targeting males aged 20 to 80 who regularly ran. The study aimed to discover physical changes and risk factors of running according to aging. Based on the findings of this study, the hypothesis was partially accepted as elderly runners showed lower physical characteristics and dynamic joint function while running compared with their younger counterparts.

**Figure A1:**
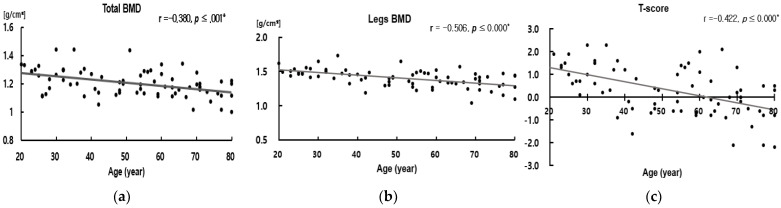

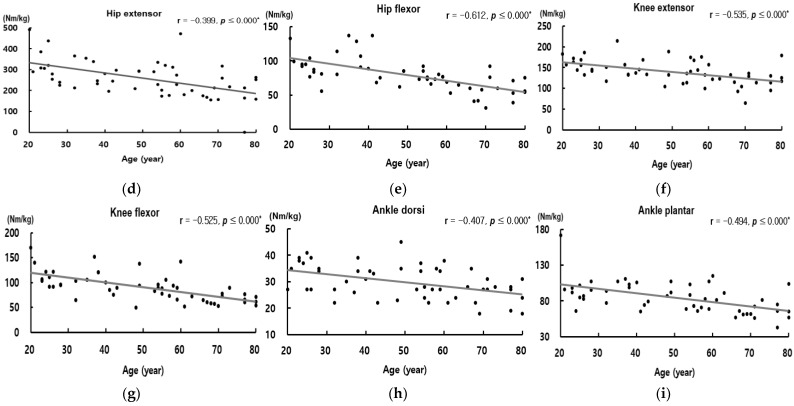
Correlations between age and physical characteristics (BMD and peak muscle torque). (**a**) Total BMD, (**b**) legs BMD, (**c**) T-scope, (**d**) hip extensor, (**e**) hip flexor, (**f**) knee flexor, (**g**) knee extensor, (**h**) ankle dorsi flexor, (**i**) ankle plantar flexor. * *p* < 0.05 indicates significant correlations between age and the variables.

When looking at the relationship between age increase and BMD and maximum strength, targeting males who regularly ran, a negative correlation was found in total BMD, legs, and T-score. A negative correlation was found in the maximum strength of the ankle, knee, and hip joints with increased age, with the power being 41.2% in the hip joint flexion power (Appendix A, Figure A1). In comparing the analysis result of the leg BMD of 2657 general elderly males and the analysis result of the older adults who regularly performed running in this study, the leg BMD of this study was 31% higher [38]. Regarding why running exercise affects BMD, this study infers that bone density decreases if stress (shock) is given to bone repeatedly by running exercise, as shown in Wolff’s law [39].

Furthermore, due to age increase, the weakening strength of the lower extremity joint displays a considerable physiological change and may become a cause of fracture injury. As for males in general, knee joint extension strength decreases by 12–15% every 10 years [33,40]. In this study, the muscle flexibility of the males who ran regularly was reduced as they aged, especially if there was a large difference in ankle joint dorsiflexion (tibialis anterior) and plantar flexion (gastrocnemius) [27]. Therefore, it is plausible that more power can be exerted because of the compensation action of the hip joint. On the other hand, when an older adult continues to exercise, the gastrocnemius and thigh skeletal muscle develop. As a result of comparing the lower extremity joint maximum strength in this study to a previous study [41], a delayed reduction in muscle strength in elderly runners was found. Regular participation in physical activities is vital in reducing physical change due to aging and the risk factors of musculoskeletal injuries [27,42]. Running exercise is a valuable method to reduce and prevent BMD. If one steadily runs, diseases and secondary fracture injuries due to bone reduction symptoms such as osteoporosis can be prevented in advance, and BMD can be retained. Additionally, running exercise can reduce the risk of fracture injuries and improve quality of life. Although the BMD volume and strength of the elderly group who continued to run were lower than those of the young adult male group, their BMD volume in the lower extremity joint was more significant than that of the elderly group who did not exercise. As age increases, the knee joint extensor muscle (thigh skeletal muscle) decreases, the iliopsoas that flexes the hip joint weakens, and the hip joint flexor muscle seems to weaken. However, regular running in the elderly would delay a rapid decrease in BMD and lower extremity muscle strength, which may be beneficial for reducing injury risk.

**Figure A2:**
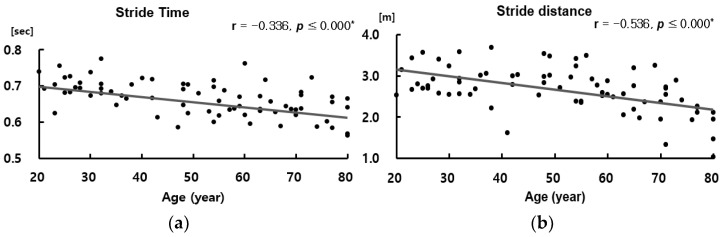
Correlations between age and impact and running characteristics. (**a**) Stride time, (**b**) stride distance. * *p* < 0.05 indicates significant correlations between age and the variables.

Upon running, the primary cause of injuries is one’s foot repeatedly touching the ground; the impact (shock) causes stress fracture, patellofemoral disorder, cartilage destruction, and lower back pain [43]. As the impact force is generated upon landing, it has been known that older adults’ injury ratio is high due to physical weakness [44,45]. A positive relationship was observed upon examining the ground reaction force variables with age, but a significant level was not meaningful after weight standardization. No statistically significant difference was revealed between the impact variables as age increases in the impulse, impact force, maximum ground reaction force, and loading rate. On the other hand, this study’s magnitude of impact variables was similar to that of the previous study [31] and, with increased age, the vertical ground reaction force becomes smaller in elderly runners. This condition is conjectured to occur because stride becomes shorter and stride frequency increases in elderly runners (Appendix A, Figure A2). However, there may be a higher risk of accumulated high magnitude of impact for elderly runners due to weaker BMD and muscle strength.

**Figure A3:**
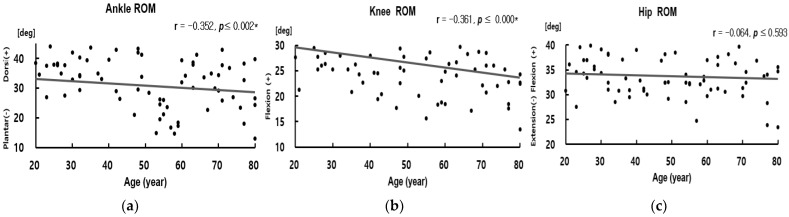

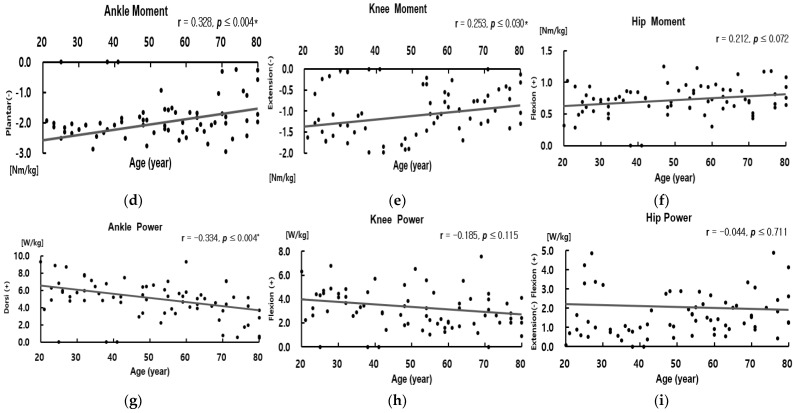
Correlations between age and lower extremity joint biomechanics upon running. (**a**) Ankle ROM, (**b**) knee ROM, (**c**) hip ROM, (**d**) ankle moment, (**e**) knee moment, (**f**) hip moment, (**g**) ankle power, (**h**) knee power, (**i**) hip power. * *p* < 0.05 indicates significant correlations between age and the variables.

The decreased range of ankle joint and knee joint motion showed an increase with age, but the hip joint range of motion showed no changes in the sagittal plane (Appendix A, Figure A3). Ankle joint range of motion is affected by aging most, and the knee range of motion becomes larger as running speed increases [30,46]. The result of this study is similar to the result of previous studies, and it was confirmed that the range of motion in the ankle joint becomes smaller as age increases [26,27,47]. Previous studies also suggested that the thigh skeletal muscle and hamstring strength weaken in elderly runners [48]. The result is linked with how the ankle joint flexor muscle negatively correlates with age. Due to reduced strength flexibility, an ankle joint sprain, and tibialis posterior and tibialis anterior injuries can be caused as the ankle joint range of motion becomes smaller. If the knee joint is not smoothly moved, the impact occurring during running is thought to be delivered to the whole body. A positive correlation between males running regularly, indicating a slowly decreased ankle joint plantar flexion moment and knee joint extension moment, was found as age increased (Appendix A, Figure A3).

A negative correlation shows slowly decreased ankle joint dorsiflexion and plantar flexion power with increased age. Additionally, a negative correlation was revealed between the knee extension power, indicating absorption of joint energy and age. The runners propel themselves forward with generations of joint power in the lower extremity during running. However, if the ankle joint’s power generating ability decreases, compensation occurs in the knee and hip joints [31,49]. On the other hand, many portions of impact absorbed in the lower extremity joints are also transferred to the whole body, so there is a relation between the level of impact of the body and the lower extremity joint angle [50]. If the load transfer is not effectively controlled, a substantial impediment is caused to the lower extremity musculoskeletal system due to repetitive high impact force [51]. The load is significantly buffered on the ankle joint and then transferred to the knee and hip joints. As age increases, the load ability of the ankle is reduced, and the risk of ankle injury may increase [52]. If enough ankle joint strength and bone intensity are not maintained in elderly runners, there is a high possibility that ankle joint injuries may occur due to impact occurring on the ground during running. As age increases, decreased dynamic joint function, showing lower joint power due to weak lower extremity joints, especially in the ankle, may compromise running performance and induce the risk of joint injuries.

Synthesizing all these, this study found that the musculoskeletal system can be maintained by running as age increases. However, the function of the lower extremity joints significantly weakens linearly, especially when the ankle joint function significantly decreases. The load of the lower extremity joint upon running is thought to be eased by muscle strength in physical strength. If one does not run correctly due to weakening strength according to aging, the physical activity can be connected to injuries, not health enhancement.

There are some study limitations that need to be addressed for the future direction of this area. First, a slight difference in biomechanical variables may exist between treadmill and overground running as this study was conducted on a treadmill. Second, the skill level and experience of the runners were not controlled among different age groups, but they may reflect different characteristics of running patterns. Finally, a longitudinal study investigating the effect of aging on physical and biomechanical changes in running may require in future research.

## 5. Conclusions

This study targeted males aged 20 to 80 who were regularly running, and it aimed to determine the relationship among physical characteristics, running variables, and age. First, the T-score of the males aged 40 to 80 who regularly ran was found to be in the normal scope. The lower extremity maximum strength decreased in hips and knees among people in their 20s and decreased in the ankle joints among those in their 60s. Specifically, a considerable decrease was found in males in their 70s in ankle joint plantar flexion. Second, the stride length in the running characteristics fell as age increased. Stride showed a difference among the groups, but stride frequency showed an increased trend as age increased. This is related to stride frequency when older adults run. Third, it was observed that the knee joint range of motion and ankle joint movement remarkably decreased in running biomechanics as age increased. When synthesizing the results, the physical characteristics gradually decreased as age increased, and the BMD and lower extremity strength of the males who regularly ran was maintained and improved compared to non-runners. Running characteristics improved if one regularly ran. Specifically, the ankle joint movements were remarkably reduced due to aging, and impact absorption was further shown on the ankle joint as age increased. As a result of examining physical characteristics and kinematic variables, the burden on the ankle joint was more evident among males in their 60s due to their weakening lower extremity joint strength. Therefore, proper running intensity and method may be applicable to prevent running-related injuries in older runners by distributing the impact and load among the lower extremity joints based on the findings. A further biomechanics study, considering the changes in physical strength and motion by aging, may suggest a more effective training method for elderly runners to maintain musculoskeletal health and ensure injury prevention.

## Figures and Tables

**Table 1:** Subject characteristics for each age group.

Group	20s	30s	40s	50s	60s	70s~80s	Total
**Number of participants**	12	12	11	12	12	16	75
**Age (year)**	24.67 ±2.46	33.83 ±2.79	45.18 ±3.38	55.42 ±2.36	64.33 ±2.99	75.13 ±3.93	51.12 ±18.04
**Height (m)**	1.75 ±0.05	1.76 ±0.04	1.72 ±0.05	1.71 ±0.04	1.67 ±0.05	1.67 ±0.06	1.71 ±0.06
**Weight (kg)**	73.58 ±5.92	74.58 ±6.11	67.64 ±6.95	66.83 ±4.02	63.42 ±7.37	62.67 ±6.09	68.07 ±7.46

**Table 2:** Correlations (R, *p*-value) between age and the variables (i.e., BMD, strength, running parameters, and running biomechanics).

**Age**	**BMD**	**Total**	**Legs**	**T score**			
		−0.380 *	−0.506 **	−0.422 *			
		0.001	0.000	0.000			
	**Strength**	**Gluteus Maximus**	**Iliopsoas**	**Quadriceps**	**Hamstring** **s**	**Tibialis Anterior**	**Gastrocnemius**
		−0.399 *	−0.612 *	−0.535 *	−0.525 *	−0.407 *	−0.494 *
		0.000	0.000	0.000	0.000	0.000	0.000
	**Running Parameter**	**Stride Time**	**Stride** **Distance**				
		−0.336 *	−0.536 *				
		0.003	0.000				
	**Impact**	**Impulse**	**Impact Force**	**GRF Peak**	**Loading Rate**		
		−0.021	0.100	0.018	0.033		
		0.864	0.417	0.881	0.784		
	**Joint Angle**	**Ankle Dorsi Flexion**	**Ankle Plantar Flexion**	**Ankle RoM**	**Knee Flexion**	**Knee Extension**	**Knee RoM**
		0.001	0.321 *	−0.352 *	−0.115	0.181	−0.361 *
		0.991	0.006	0.002	0.335	0.129	0.000
		**Hip Flexion**	**Hip Extension**	**Hip RoM**			
		0.164	0.289 *	−0.064			
		0.166	0.013	0.593			
	**Joint Moments**	**Ankle Dorsi Flexion**	**Ankle Plantar Flexion**	**Knee Flexion**	**Knee Extension**	**Hip Flexion**	**Hip Extension**
		0.328 *	−0.177	0.253 *	−0.166	0.212	0.225
		0.004	0.140	0.030	0.167	0.072	0.060
	**Joint Power**	**Ankle Absorption**	**Ankle Generation**	**Knee Absorption**	**Knee Generation**	**Hip Absorption**	**Hip Generation**
		−0.334 *	0.326 *	−0.185	0.357 *	−0.044	0.082
		0.004	0.006	0.115	0.002	0.711	0.494

BMD: bone mineral density, GRF: ground reaction force. * *p* < 0.05 indicates significant correlations between age and the variables. ** *p* < 0.01.

## Data Availability

Not applicable.
